# Reforming the white coat economy: judicial evidence and institutional implications from China’s healthcare anti-corruption campaign

**DOI:** 10.3389/fpubh.2026.1818452

**Published:** 2026-05-18

**Authors:** Zhiling Shen, Jiao Guo, Jiao Li, Zhiyu Chen, Ying Tang

**Affiliations:** 1 Discipline Inspection and Supervision Office, Chongqing Medical and Pharmaceutical College, Chongqing, China; 2 School of Marxism, Chongqing Medical and Pharmaceutical College, Chongqing, China; 3 Discipline Inspection Office, Chongqing Traditional Chinese Medicine Hospital, Chongqing, China; 4 School of Chemistry and Chemical Engineering, Chongqing University of Science and Technology, Chongqing, China; 5 Discipline Inspection and Supervision Group of the Chongqing Municipal Commission of Discipline Inspection and Supervision stationed at Chongqing Municipal Health Commission, Chongqing, China

**Keywords:** adjudicative document analysis, anti-corruption in healthcare, healthcare corruption, judicial evidence, legal mechanisms, white coat economy

## Abstract

**Objective:**

This study aimed to construct a reproducible sample of judicially visible healthcare corruption in China, identify its typologies, risk nodes, actor structures, and elucidate the institutional mechanisms underlying representative cases.

**Methods:**

An explanatory sequential mixed-methods design was used. Criminal judgments with dates from 1 September 2019 to 31 December 2025 were retrieved from China Judgments Online in January 2026 and screened, yielding 573 judgments involving 787 defendants. Phase 1 used structured coding for descriptive analysis, while phase 2 used embedded multiple-case analysis of purposively selected, information-rich cases to explain key institutional mechanisms.

**Results:**

Cases clustered into three main categories, namely pharmaceutical/clinical commercial bribery (267, 46.60%), embezzlement of in-hospital funds (137, 23.91%), and medical insurance fund fraud (99, 17.28%). Risk points concentrated on procurement (335 cases), medical insurance auditing/payment (136 cases), and in-hospital finance/asset management (122 cases). Among defendants sentenced to imprisonment (*n* = 710), the median principal penalty was 36 months, probation was 42.90%, fines was 91.70%, and recovery/disgorgement/forfeiture was 84.81%. Cross-case analysis revealed that corruption hinged on the coupling of gatekeeping authority, supplier inducement, professional discretion, and control over payment or accounting interfaces.

**Conclusion:**

Publicly available criminal judgments provide a reproducible risk map of judicially visible healthcare corruption in China. Findings support node-oriented governance prioritizing procurement transparency, payment-side auditing, and high-risk internal posts. These data should be interpreted as legally filtered governance evidence rather than as direct estimates of underlying corruption incidence.

## 1 Introduction

The “white coat economy” refers to rent-seeking around entrusted powers in healthcare, including prescribing, procurement, and insurance settlement. The manifestations of this economy encompass commercial bribery, procurement corruptions, informal payments, medical insurance frauds, and embezzlements, often involving strategic interaction and collusion among healthcare institutions, supply-chain actors, payers, and regulators (1–5). Health-sector corruption can distort resource allocation, compromise care quality, and erode trust and equity. International research has linked corruption to reduced medical trust and inequitable access, especially in systems with high resource intensity and long principal–agent chains (4, 6, 7). However, research faces three challenges, namely unclear conceptual boundaries, limited observable data, and poor comparability across countries and institutional settings. These constraints require reproducible observational strategies within clear typologies and a distinction between judicial records and underlying corruption (1–3).

In China, healthcare corruption governance has evolved amid institutional reform. Pharmaceutical procurement, medical service delivery, and medical insurance management involve high capital intensity, professional barriers, and multiple supervisory actors, creating conditions for collusion. Legally processed cases typically involve commercial bribery, embezzlement, and insurance fraud, spanning healthcare personnel, administrators, and supply-chain actors (5). Policy and institutional studies further suggest that repeated governance rounds since 2013 have sought to curb rent-seeking through tighter rules. In September 2019, China expanded centralized drug procurement to reshape payment–procurement relations and compress rent-seeking space (8). Whether these reforms have altered the structure and mechanisms of healthcare corruption remains an open question (9).

Since 2023, China’s healthcare anti-corruption campaign has intensified, with high-profile enforcement extending into 2025 (10–14). Multiple authorities have strengthened the regulation of pharmaceutical procurement, hospital governance, and insurance fund supervision. Publicly disclosed criminal judgments provide a window into how corruption risks are distributed across procurement, clinical, and payment-related nodes. Despite growing policy attention, three empirical gaps remain. First, most existing studies focus on 2013–2019 or use small samples, limiting observation of recent structural changes. Second, adjudicative documents face temporal and regional variation in disclosure, which may bias descriptive interpretation if not addressed. Third, existing research has yet to translate corruption typologies into operational governance-risk mapping for precise institutional diagnosis (15, 16).

Against this background, this study adopted an explanatory sequential mixed-methods design to describe judicially visible healthcare corruption and explain institutional mechanisms in representative cases. The study focused on three questions, namely distribution of case types, actors, and nodes; adjudicative patterns; and governance implications.

## 2 Methods

### 2.1 Research design

**Figure 1:**
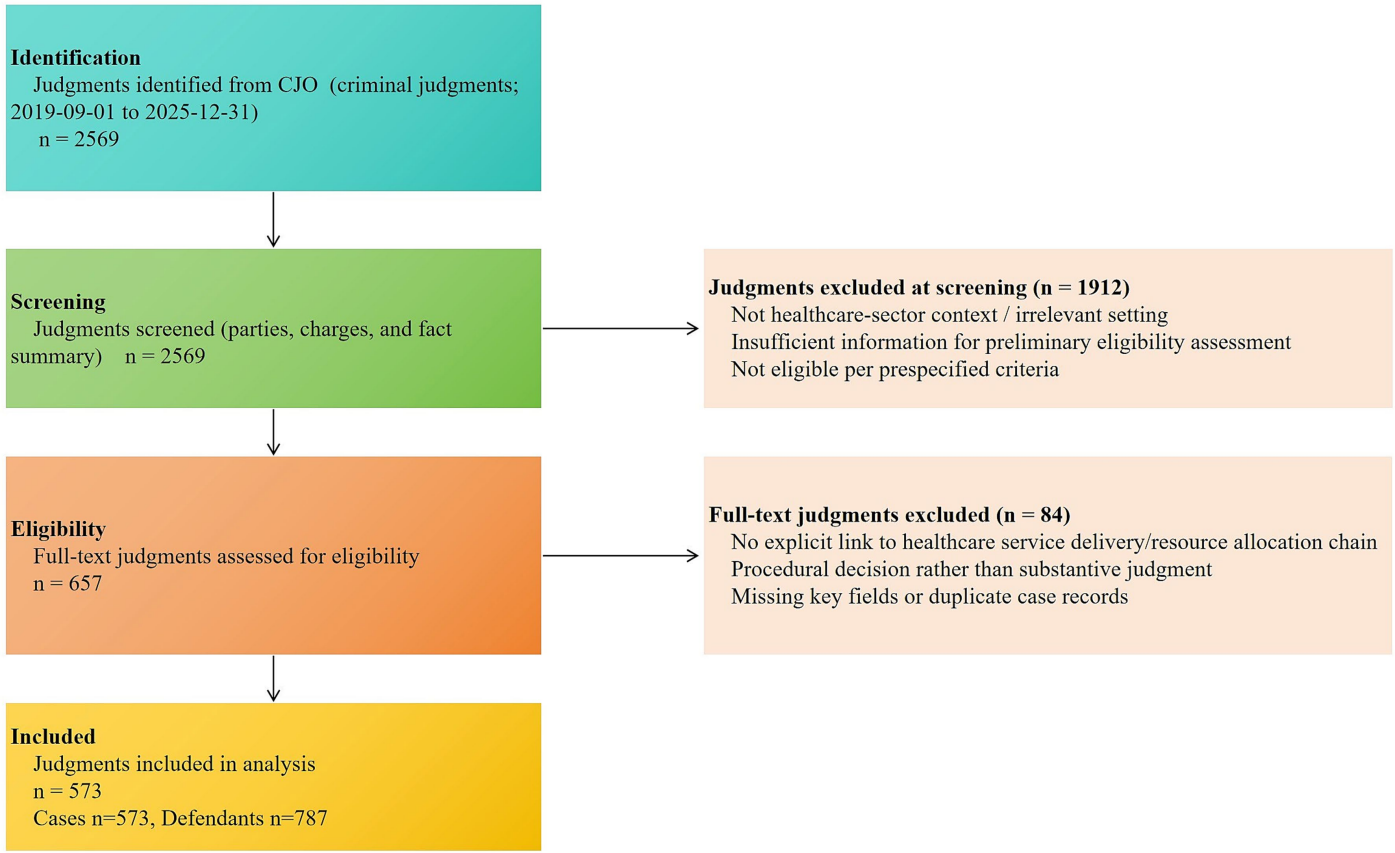
Identification, screening, eligibility assessment, and inclusion of CJO criminal judgments related to healthcare-sector corruption, September 2019–December 2025.

This study adopted an explanatory sequential mixed-methods design. In phase 1, adjudicative documents were analyzed for descriptive and distributional patterns of judicially visible healthcare corruption cases with judgment dates between 1 September 2019 and 31 December 2025. Because judgment year does not align with the timing of corrupt conduct, annual counts were treated descriptively rather than as causal trend estimates (17, 18). September 2019 was chosen as the starting point, marking the expansion of national centralized drug procurement, a policy shift that was likely to reshape corruption risks in pharmaceutical distribution (5, 8, 19). In phase 2, embedded multiple-case analysis was conducted to examine institutional enforcement mechanisms, circumvention strategies, and chained risk evolution not fully captured in phase 1. Integration and reporting followed established principles for mixed-methods quality and integration in health services research (17, 20–24) (see Figure 1).

### 2.2 Data sources

This study used criminal adjudicative documents from China Judgments Online (CJO) in January 2026, with judgment dates between 1 September 2019 and 31 December, 2025, as the primary data source. Since 2014, Chinese courts have centrally released large numbers of adjudicative documents on CJO, making it a relatively high-density source for legal-text research. However, documents on CJO do not constitute the complete set of healthcare corruption cases, as disclosure may vary across courts and over time and may be affected by selective disclosure. Accordingly, the dataset should be regarded as a sample of publicly disclosed judicial judgments rather than a comprehensive account. Retrieval records and metadata were retained for reproducibility, and all cases were de-identified prior to reporting.

### 2.3 Case identification and retrieval strategy

Because CJO lacks a stable, verifiable subject tag for “healthcare corruption,” this study used a three-dimensional retrieval framework combining charges, healthcare context, and transaction pathways to balance recall and precision. Specifically, the strategy included: (1) fixed field restrictions (such as criminal cases, judgments, and judgment dates from 01 September 2019 to 31 December 2025); (2) a set of charges strongly associated with healthcare corruption and subject to criminal law, such as bribery, embezzlement, misappropriation, encroachment, medical insurance fund fraud, and bid rigging, as the legal entry point; and (3) full-text searches using healthcare institutional context terms (e.g., hospitals, health centers, medical insurance, pharmaceuticals, and consumables) and transaction pathway terms to identify texts within the healthcare service–resource allocation chain involving benefit transfer or fund infringement centered on entrusted powers. The search formulas, keyword dictionaries, execution steps, and retrieval log templates are provided in Supplementary Appendix S1. Formal eligibility assessment was conducted during subsequent sample review.

### 2.4 Sample deduplication and eligibility review

After merging the retrieval results, screening and review proceeded in three stages. First, duplicates were removed using a minimal unique key composed of case number, adjudicating court, and judgment date. Suspected duplicates were manually verified using defendant information, charges, and key facts, and the more complete version was retained. Second, clearly irrelevant texts were excluded through preliminary screening of titles, causes of action, and fact summaries. Third, two researchers independently reviewed full texts for eligibility. Cases were included when (1) adjudicative facts occurred within the healthcare service-resource allocation chain, including procurement, clinical service, medical insurance settlement/fund supervision, or in-hospital financial/asset management; (2) the conduct was confirmed by a criminal judgment and met this study’s operational definitions of corruption, fraud, or collusion; and (3) the text provided sufficient information for core variable extraction. Structured extraction and coding were conducted using Microsoft Excel with data validation, filtering, and pivot tables. A third researcher independently reviewed coding and spot-checked core variables, including type, subject role, occurrence node, and adjudicative outcome. Change records and version numbers were retained for auditability and reproducibility.

### 2.5 Statistical analysis

Data organization, summarization, and statistical analysis were performed using Microsoft Excel. Descriptive statistics used pivot tables to summarize case and defendant counts, type structure, subject roles, and adjudicative indicators by month, quarter, and year, along with stratified comparisons by region and court level. All coding sheets, dictionaries, rules, and manual changes were maintained under version control to ensure traceability from original texts to analytical data. No causal time-series model was fitted; annual variation was used only for descriptive comparison, given publication delays and constraints on selective disclosure.

### 2.6 Multiple case analysis

To build on quantitative findings and formulate actionable governance diagnoses, the second phase used an embedded multiple-case analysis to examine institutional enforcement mechanisms, circumvention strategies, and chained risk evolution (20). Cases were purposively selected from the phase 1 sample, with individual judgment texts and associated public materials as the unit of analysis. Priority was given to variation in corruption type, subject combination, and region to support mechanism comparison. For each case, an evidence package was constructed containing key points from the judgment’s factual findings and reasoning, involved nodes and role chains, and public text evidence on corresponding regulatory rules and process nodes (e.g., bidding and procurement, insurance settlement and auditing, and in-hospital financial and asset management). Cross-case analysis used pattern matching and explanatory synthesis to identify recurrent mechanisms, circumvention strategies, and regulatory gaps. These findings were then interpreted alongside phase 1 structural results to generate governance implications for procurement, clinical practice, and medical insurance payment.

## 3 Results

### 3.1 Analysis of adjudicative documents

#### 3.1.1 Sample acquisition and inclusion process

Following the retrieval strategy in Supplementary Appendix S1, this study applied a one-judgment-per-case rule, retaining only the final effective judgment for each case. After retrieval and deduplication, 2,569 candidate documents were identified. Preliminary screening based on party identities and fact summaries excluded 1,912 texts that were clearly unrelated to the healthcare service–resource allocation chain or lacked sufficient information, leaving 657 documents for full-text review. Intensive reading excluded another 84 texts because the facts lacked a clear connection to the healthcare service–resource allocation chain, the documents were procedural rulings rather than substantive judgments, or key fields were missing or duplicated. Ultimately, 573 criminal judgments involving 787 defendants were included as the final sample.

As shown in Table 1, publicly available judgments exhibited a marked trough during 2021–2023 (58, 20, and 21 cases, respectively). This pattern should not be interpreted as a decline in underlying corruption, but rather as the combined effect of procedural delay, uneven CJO disclosure, and pandemic-related disruption. By contrast, the intensified 2023 anti-corruption campaign is expected to appear with delay, more often in 2024–2025 than in 2023 itself.

**Table 1:** Basic characteristics of the sample, types of corruption, and governance risk points.

Indicators	*n*	%
A. Time distribution (by judgment year)	573	100%
2019 (Sep–Dec)	158	27.57%
2020	180	31.41%
2021	58	10.12%
2022	20	3.49%
2023	21	3.66%
2024	57	9.95%
2025	79	13.79%
B. Region	573	100%
Eastern China	131	22.86%
Central China	188	31.81%
Western China	205	35.78%
Northeast China	49	8.55%
C. Court level	573	100%
Primary court	534	93.19%
Intermediate court	37	6.46%
High court	1	0.17%
Specialized court	1	0.17%
D. Main types of healthcare corruption (mutually exclusive primary categories)	573	100%
Pharmaceutical sales/clinical commercial bribery	267	46.60%
Embezzlement of in-hospital funds (corruption/embezzlement/misappropriation of funds)	137	23.91%
Fraud (false reimbursement of medical insurance funds, impersonation)	99	17.28%
Bribery of non-state functionaries/bribery to non-state functionaries/corporate bribery	45	7.85%
Bid rigging	25	4.36%
E. Governance risk points (multiple selection possible, counted based on whether the case involves this link)	—	—
Procurement and supply chain (pharmaceuticals/consumables/equipment/informatization/construction/business cooperation and outsourcing, etc.)	335	—
Medical insurance audit and payment (settlement/audit/inspection/impersonation, etc.)	136	—
In-hospital finance and asset management (budget, billing, reimbursement, bills/notes, asset disposal, etc.)	122	—
Administrative supervision and resource allocation (access qualification/fund allocation, personnel management, etc.)	61	—

Cases may involve multiple governance risk points; therefore, the sum of counts across risk points exceeds the total number of cases (*n* = 573).

Regionally, judgments were concentrated in western China (205, 35.78%) and central China (188, 31.81%), followed by eastern China (131, 22.86%) and northeastern China (49, 8.55%). These differences likely reflect healthcare system size, procurement structures, enforcement intensity, and uneven court publication, and should be interpreted as judicial visibility, not as a ranking of underlying corruption incidence.

#### 3.1.2 Typological spectrum and governance risk points

Among the 573 cases (Table 1), primary case types were highly concentrated. Pharmaceutical procurement and sales, together with clinical commercial bribery, accounted for the largest share (267, 46.60%), followed by embezzlement of in-hospital funds, including misappropriation, embezzlement, and encroachment (137, 23.91%), and medical insurance fund fraud (99 cases, 17.28%). These three categories together comprised approximately 88% of all cases. The remaining categories included bribery of non-state functionaries/unit bribery (45, 7.85%) and bid rigging (25, 4.36%).

When mapped onto governance risk points, risk is concentrated around three high-frequency nodes, namely procurement and supply chain management (335 cases), medical insurance auditing and payment (136 cases), and in-hospital financial and asset management (122 cases). Fewer cases involved administrative supervision and resource allocation, such as access qualifications, fund allocation, or personnel management (61 cases). Overall, judicially visible corruption risk clustered at nodes where resources enter and funds exit healthcare institutions.

To examine selective disclosure effects, this study stratified the sample by judgment year, court level, and region. By year, pharmaceutical/clinical bribery ranged from 38.1% (2023) to 50.0% (2019). By court level, bribery accounted for 46.25% in primary courts and 51.35% in intermediate courts. Regionally, commercial bribery dominated Central China (44.7%) and Western China (62.4%), while in-hospital fund embezzlement was slightly higher in Eastern China (32.82% vs. 30.53%) and Northeast China (36.73% vs. 30.61%). The sharp decline in case volume during 2021–2023 reflects changes in judicial disclosure policies rather than shifts in corruption patterns (15). These checks indicate that the core typological findings are robust to sample selection biases inherent in publicly available adjudicative documents.

#### 3.1.3 Key actors and transaction pathways

As shown in Table 2, panel A classifies defendants by role, while panel B identifies the individuals within the healthcare system who held entrusted power and were targeted by external defendants (e.g., suppliers and third-party service providers).

**Table 2:** Key actors and transaction pathways.

Indicators	*n*	%
A. Actors involved (defendants)	787	100%
Hospital leadership (presidents/party secretaries/deputy heads)	223	28.34%
Third-party service providers (suppliers of medical devices, pharmaceuticals, consumables; construction and engineering contractors, etc.)	141	17.92%
Personnel in key positions (accountants, billing clerks, cashiers, etc.)	114	14.49%
Heads of administrative/functional departments (finance/procurement/equipment/logistics, etc.)	99	12.58%
Clinical medical and nursing staff	88	11.18%
Heads of clinical/medical technology departments	59	7.5%
Health administration officials and heads of relevant state-owned enterprises/institutions	45	5.72%
Others (patients/intermediaries/specified related persons)	14	1.78%
Hospitals and their internal departments (as institutional actors)	4	0.51%
B. Targets of external defendants (actors with entrusted power)	145	100%
Hospital leadership (presidents/party secretaries/deputy heads, including those at township health centers and private hospitals)	65	44.83%
Heads of administrative/functional departments (finance/procurement/equipment/logistics, etc.)	40	27.59%
Heads of medical technology/clinical departments	19	13.10%
Clinical medical and nursing staff	12	8.28%
Health administration officials and heads of relevant state-owned enterprises/institutions	7	4.83%
Personnel in key positions (accountants, billing clerks, cashiers, etc.)	2	1.38%
C. Transaction pathways (top 3)	—	—
Cash/bank transfers/real estate/vehicles/gifts & cash equivalents	272	—
Kickbacks/rebates/commissions	116	—
Fraudulent contracts/disguised dividends/disguised payments (“training fees,” “service fees,” “interest,” “loans,” etc.)	53	—

Panel A classifies defendants by their organizational role. Panel B identifies the targets of corruption initiated by external defendants (e.g., suppliers and third-party service providers); these targets are individuals with entrusted power within the healthcare system.

Among 787 defendants, involvement clustered into three groups, namely hospital leadership (223, 28.34%), third-party service providers (141, 17.92%), and key position personnel (accountants, billing clerks, and cashiers) (114, 14.49%). Together, these three groups accounted for 60.74% of all defendants, indicating corruption centered on nodes influencing resource allocation, fund movement, or external transactions. Remaining defendants included administrative/functional department heads, clinical staff, clinical/medical technology heads, and health administration officials (Table 2A).

Among the targets of external defendants (*n* = 145), authority concentrated in few positions, namely hospital leadership (65, 44.83%) and administrative/functional department heads (40, 27.59%), together accounted for 72.41%; including medical technology/clinical department heads (19, 13.10%) raised the share to 85.52% (Table 2B). This pattern suggests that entrusted authority, as reflected in judicial texts, concentrated in decision-making, approval, allocation, and resource control positions.

Among identifiable transaction pathways (*n* = 441), the most common were cash, bank transfers, real estate, vehicles, gifts, and equivalents (272, 61.68%), followed by kickbacks/rebates/commissions (116, 26.30%), and disguised payments (e.g., “training fees,” “service fees,” “interest,” or “loans”) (53, 12.02%) (Table 2C). These findings indicate that both direct monetary transfers and more concealed contractualized forms remained important channels of benefit transfer.

#### 3.1.4 Judicial dispositions and adjudicative characteristics

Defendant-level outcomes showed a concentrated pattern of principal penalties, combined with frequent ancillary sanctions and leniency factors. The median principal penalty was 36 months (IQR 30). Fines were imposed on 91.70% of defendants, and recovery/confiscation on 84.81%. Guilty plea or acceptance of punishment was recorded in 81.43%, and voluntary surrender, meritorious service, or frank confession was recorded in 89.73% (Table 3).

**Table 3:** Judicial dispositions and discretionary factors.

Primary case type	Defendants (*n*)	Median principal penalty (months)	Probation (%)	Fine (%)	Recovery/disgorgement/forfeiture (%)	Guilty plea and acceptance of penalty (%)	Voluntary surrender/meritorious service/frank confession (%)
Bribery (active and passive)	268	36 (30)	28.73%	96.64%	83.58%	85.07%	94.4%
Fraud	202	36 (23)	61.39%	100%	84.65%	75.74%	86.14%
Misappropriation	173	36 (38)	28.9%	74.57%	92.49%	78.03%	87.86%
Bid rigging	30	9.5 (5.75)	86.67%	100%	70%	86.87%	100%
Commercial bribery (non-state actor)/bribery of non-state functionaries	34	17.5 (13.75)	79.41%	76.47%	79.41%	91.18%	85.29%
Corporate bribery	3	30 (10)	33.33%	100%	0	100%	66.67%
Total	710	36 (30)	42.90%	91.70%	84.81%	81.43%	89.73%

This table is based on defendants sentenced to fixed-term imprisonment (*n* = 710). Defendants not sentenced to fixed-term imprisonment (e.g., sentenced to criminal detention, public surveillance, exempted from criminal punishment, or subject only to fines) are excluded from this table.

Across primary case types, the median principal penalty was similar for bribe-taking/giving, embezzlement/misappropriation/encroachment, and medical insurance fraud (all 36 months), but the timing of sentence execution differed. Probation was less common in bribe-taking/giving (28.73%) and embezzlement (28.90%) than in insurance fraud (61.39%). Bid rigging cases received lighter sentences, with median principle penalty of 9.5 months, probation granted in 86.67%, and fines imposed in 100%. Bribery involving non-state functionaries had a short- to-medium-term penalty (median 17.5 months) and high probation (79.41%). Overall, among categories with similar principal penalties, differences in probation, pecuniary sanctions, and leniency factors accounted for most sentencing variation (Table 3).

### 3.2 In-depth depiction of typical cases

#### 3.2.1 The institutional-gatekeeper type

The institutional-gatekeeper type is characterized by actors who control organizational entry points (e.g., hospital leadership and department heads) and convert personal or group preferences into institutional rules and procedures, thereby giving corruption a compliant appearance and making it durable and repeatable. In the cases analyzed, this type was concentrated in decision-making, procurement rule-setting, financial categorization, and compensation arrangements.

One recurring pathway was the capture of clinical procedures to create off-book fund pools. In grassroots settings, funds from intercepted fees, fraudulent invoicing, and drug kickbacks were pooled and redistributed under compliant labels (e.g., “welfare” and “bonuses”), with extra shares to decision-makers. In one case, this included division of surplus operating funds, illegal distribution of official-account funds, and drug kickbacks that increased from 5% to 10%. The court also identified additional distributions to ‘meeting members’ totaling a six‑figure RMB sum (Supplementary Appendix S2, Case 1). This illustrates how institutional deliberation and categorization can convert fragmented misconduct into a stable organizational interest structure.

A second pathway involved capturing source rules for long-term rent extraction. In equipment procurement, corruption occurred not at bid evaluation but earlier, during the formulation of technical parameters and tender documents. In one case (Supplementary Appendix S2, Case 2), a procurement valued at a multi‑million RMB sum generated an immediate six‑figure cash payment, while the same mechanism later enabled the supplier to win contracts totaling an eight‑figure RMB sum, demonstrating how early‑stage rule capture can produce sustained rents.

A third pathway was internal monetization of organizational resources through control over rulemaking and interpretation. In one compensation case, actors used internal documents to establish formal identities and unilaterally adjust pay standards, enabling an individual to receive high compensation for 7 years without performing duties, with cumulative embezzlement of a six-figure RMB sum (Supplementary Appendix S2, Case 3). In another case, drug and consumable rebates totaling an eight-figure RMB sum were absorbed as a shadow budget under labels such as “net profit awards” and “consultation fees,” alongside abuse of power resulting in state losses of a seven-figure RMB sum and secondary collective embezzlement (Supplementary Appendix S2, Case 4). Together, these cases demonstrate how the institutional-gatekeeper type converts individual violations into organizationally reproducible arrangements through process embedding, categorical packaging, and group-based redistribution.

#### 3.2.2 The external supplier type

The external supplier type follows supply-side inducement and organizational internalization. Market actors competing for scarce institutional resources (e.g., formulary access and project awards) use repeated, often nominally concealed transfers to cultivate in-hospital gatekeepers–department heads, procurement personnel, and hospital leaders–rather than one-off payments. Through temporal dispersion and nominal concealment, they reduce visibility and stabilize exchange.

In the pharmaceutical supply chain, this mechanism often takes an organizational form. In one case, a supplier made 73 cash payments totaling 3 million RMB over 10 years to pharmacy directors at two hospitals, securing favor for formulary access and drug replacement. This case included 14 payments totaling 2 million RMB to one director and 59 payments totaling 1 million RMB to another; the court identified these as organized conduct reflecting corporate intent (Supplementary Appendix S2, Case 5). Sustained supplier investment thus converted external competition into a predictable bias within hospital decision-making.

The same mechanism appeared in construction and project bidding. A contractor cultivated a hospital director and spouse with low-visibility gifts over 8 years (such as New Year visits and birthdays), totaling a six-figure RMB sum, before winning an interior decoration contract worth nearly forty million RMB (Supplementary Appendix S2, Case 6). Thus, the external supplier type goes beyond a simple bribe dyad, as suppliers provide sustained inducement, while in-hospital actors convert transfers into outcomes through access, bidding, or formulary decisions. Cross-case comparison indicates this type often operates with the institutional gatekeeper type.

#### 3.2.3 The funds-handler type

The funds-handler type centers on simultaneous control over financial interfaces (such as account entry, settlement, and transfer) and evidentiary interfaces (such as bills, seals, system records, and vouchers). This dual control allows sustained appropriation at relatively low relational cost while reducing detectability by concealing the evidentiary chain. Where cash receipts and reconciliations lack rigid verification, key posts can convert routine weaknesses into durable opportunities.

In one case, a branch cashier forged seals and deposit records to divert collected funds, embezzling a seven-figure RMB sum from rental income, utility fees, and patient charges (Supplementary Appendix S2, Case 7). In this case, control over collection, deposit, and recording was concentrated in one position, enabling concealment within the same operational interface. On the medical insurance payment side, the funds handler type often involves an organized division of labor combining fraud, settlement transfer, and evidentiary concealment. In another case, the controller of a private designated institution organized 6 years of practices (such as fake admissions, falsified records, inflated days, split admissions, excessive treatment, irregular billing, and off-label drug use), defrauding the insurance fund. The audit found that falsely claimed pharmaceuticals alone accounted for a six-figure RMB sum (Supplementary Appendix S2, Case 8). This case shows that when settlement authority, fund accounts, and documentary control are concentrated in a few positions, financial gain and evidentiary resistance can be produced simultaneously.

System vulnerabilities may reinforce this pattern. In one case, a billing staff member exploited a system weakness allowing improper payment-method selection, recording cash payments as “check payments” and intercepting a substantial amount of cash, the bulk of which remained outstanding for more than three months (Supplementary Appendix S2, Case 9). This highlights that where automatic reconciliation and anomaly detection are weak, electronic records themselves can become concealment tools. Overall, the funds-handler type constitutes a durable internal closed loop by combining control over account entry, settlement, transfer, documentation, and system records.

#### 3.2.4 The technical adjudicator type

The technical adjudicator type operates within the discretionary space created by professional qualifications, technical operation rights, and information asymmetry in clinical and technology processes. Unlike institutional gatekeepers, it does not depend on formal approval authority; instead, it converts prescription, testing, consumable selection, coding, and reimbursement records into rent-seeking opportunities. One common form is prescription- or utilization-based kickback distribution. In one case, a chief pharmacist provided prescription statistics to distributors, who then distributed kickbacks to physicians based on prescription volume. Over 3 years, the actors received over 1 million RMB, with the majority going to physicians (Supplementary Appendix S2, Case 10). Those controlling prescription data and drug rules, together with payment coordinators, turned corruption into networked collaboration.

A second form is the privatized monetization of medical technology operating rights. An MRI physician bypassed the hospital billing system, privately charging for over a thousand body parts, collecting several hundred thousand RMB, and causing nearly six hundred thousand RMB in lost revenue (Supplementary Appendix S2, Case 11). This mechanism hinges on control over service availability and charging at the technical-execution level, rather than procurement or finance.

A third form couples clinical selection power with supply-chain incentives. In high-value consumables, a specialty director received 1.4 million RMB in kickbacks for favoring a specific orthopedic instrument brand (Supplementary Appendix S2, Case 12). In another case, individuals responsible for pharmacy-entrustment operations received disguised monthly payments as “consultation fees” (Supplementary Appendix S2, Case 13). Thus, technical adjudication operates through prescription discretion, access arrangements, and outsourced relations under a compliant facade.

Finally, the technical adjudicator type may couple with the payment-side data chain. In one insurance fraud case, the head of the medical insurance department knowingly uploaded false data, prepared reimbursement materials, and received commissions tied to reimbursement amounts (Supplementary Appendix S2, Case 14). Multiple clinicians implemented “yin-yang prescriptions” and assisted reimbursement, involving defrauded amounts ranging from hundreds of thousands to over one million RMB. This suggests that clinical recording and coding are not neutral technical acts, but manipulable interfaces for payment-side arbitrage when linked to reimbursement rules.

## 4 Discussion

This study reveals that judicially visible healthcare corruption in China is concentrated in a limited set of case types, actors, and governance nodes (Table 4). The dominant case categories were pharmaceutical and clinical commercial bribery, embezzlement of in-hospital funds, and medical insurance fraud, while the highest-risk nodes were procurement and supply chain management, medical insurance auditing and payment, and in-hospital financial and asset management. Taken together, these findings suggest that corruption is most likely to emerge where entrusted authority, professional discretion, external transactions, and fund-flow control converge.

**Table 4:** Typology of key actors in healthcare corruption.

Type	Scope of subjects	Basis of authority	Direction of acts	Primary areas involved	Essential characteristics
Institutional gatekeepers	Hospital leadership, heads of administrative/functional departments, health administration personnel	Statutory authority, organizational delegation	External approval/internal management	Resource allocation arena (procurement initiation/budget approval/personnel appointment/rule-making)	Highly concentrated decision-making power, broad discretionary scope, procedural constraints primarily dependent on internal processes
External suppliers	Medical device/pharmaceutical/consumables suppliers, third-party service providers, patients, intermediaries	Market position, commercial contract	Transfer of benefits into the institutional system	Market-institution interface arena (bidding/supply/agent/contracting)	Transacting parties operate under distinct logics—institutional sector pursues compliance and stability, market sector pursues share and profit
Funds handlers	Personnel in key positions (accountants, cashiers, billing clerks, etc.)	Division of functions; operational authorization; control of accounting/documentary interfaces	Internal appropriation; concealment through settlement and documentary manipulation	Billing, cashier, reconciliation, invoice verification, settlement transfer	High-frequency repetitive transactions, evidentiary concealment, dependence on system-control weaknesses
Technical adjudicators	Heads of clinical/medical technology departments; clinical medical and nursing staff	Professional knowledge; clinical discretion; control over coding/recording/selection	Internal adjudication; internal–external collusion through treatment plans or consumable choice	Prescribing, consumable selection, surgical determination, admission indication, reimbursement coding	High professional barriers, discretion embedded in clinical logic, and weak external verifiability

In China, recent procurement reforms may have narrowed visible rent-seeking while shifting misconduct toward concealed sub-nodes (e.g., settlement, coding, and auditing) (25–31). However, adjudicative documents incompletely capture informal payments and are shaped by selective disclosure and regional publication variation. Thus, observed regional concentration reflects judicial visibility rather than true incidence (32). Recent Chinese studies on medical damage disputes similarly confirm that judicial materials are legally filtered and not population-based (33, 34). This echoes international research identifying procurement, prescribing, and reimbursement as high-risk points shaped by information asymmetry and concentrated fund flows (20, 35–37).

The node-concentrated pattern identified in this study reflects a bottleneck-rents structure. Corruption clusters at mandatory passage points where discretion is high, information asymmetry is strong, external visibility is weak, and transactions are easily monetizable (1, 38). Corruption stems less from isolated moral failure than from gatekeepers’ control over scarce resources and rule-entry points, where a single decision may affect procurement volumes or reimbursement flows, enabling repeated rent extraction (39). Healthcare systems’ professional opacity and information asymmetry further expand the opportunistic space (40). Thus, the observed concentration reflects an opportunity structure produced by organizational power, transaction availability, and evidentiary traceability (35).

More specifically, three interacting mechanisms appear to drive this concentration. First, procurement nodes combine concentrated decision-making with repeated interactions, allow suppliers to influence technical parameters and bid rules, converting one-off bribery into process-based rule capture (41–44). Second, clinical decision-making links professional judgment, prescription volume, and commercial incentives, while weak external verification makes medication choices vulnerable to gifts or implicit kickbacks (45–49). Third, insurance auditing and in-hospital financial management serve as key fund-outflow gates. When settlement and accounting control are concentrated, covert transfers occur through split payments, fabricated items, or contractualized arrangements designed to reduce detectability (20). Overall, corruption at these nodes reflects a structural outcome of concentrated process control, professional opacity, and selective visibility under existing supervision.

These findings also contribute to abolitionist public health by shifting the interpretation of healthcare corruption away from a purely punitive or individualizing frame. Abolitionist public health does not simply ask how harmful acts should be punished after they occur; it asks how institutions can dismantle the social, economic, and organizational conditions that repeatedly produce harm, extraction, and preventable loss (50–52). From this perspective, the value of judicial evidence is not that it justifies an ever-expanding carceral response, but that it makes otherwise hidden institutional pathways visible. These findings show that corruption in healthcare is not merely a series of isolated moral failures. Rather, corruption is reproduced through bottleneck positions, opaque procurement rules, supplier dependence, professional discretion, weak payment-side verification, and accounting interfaces that permit public resources to be converted into private gain. This extends abolitionist public health conversations beyond policing and incarceration by showing how judicial archives can be repurposed as structural diagnostic evidence for health-system reform. The central implication is therefore preventive and institution-building, as reducing reliance on ex post criminal punishment requires earlier redesign of procurement, reimbursement, conflict-of-interest management, internal financial controls, and participatory oversight mechanisms, so that patients, public funds, and trust are protected before corruption becomes judicially visible.

These findings suggest four governance priorities, namely strengthening internal controls for high-risk posts, tightening upstream procurement governance, reinforcing supplier-side compliance, and improving payment-side auditing. In Table 2A, four high-risk internal categories—hospital leadership, administrative/functional heads, key position personnel, and clinical/medical technology heads—accounted for 469 of 787 defendants (59.59%). At the case level, risk clustered around procurement (335 cases), insurance auditing/payment (136 cases), and in-hospital finance/asset management (122 cases) (Table 1E). Accordingly, monitoring should prioritize role segregation and rotation audits for key posts; independent parameter review, scrutiny of single-source procurement, and vendor-concentration alerts; conflict-of-interest disclosure and medical representative filing verification; and anomaly-triggered re-review, payment registration, and illicit gain recovery in reimbursement settings. These are operational metrics, not statutory thresholds, and should be adapted to the hospital context. More broadly, the anti-corruption campaign should be understood as part of a state commitment to public-sector accountability and healthcare integrity.

Although stable case-linked triangulation with administrative datasets was not feasible, the node distribution aligns with external enforcement signals. National prosecutorial reporting highlights medical insurance fraud, and 2024 flying inspections focused on designated institutions, pharmaceutical procurement, and irregular fund use (13, 29–31). These priorities correspond to the payment-side, procurement, and supplier nodes in the study sample. This consistency provides contextual corroboration that the observed concentration reflects broader regulatory concern rather than publication artifacts.

## 5 Conclusion

Drawing on 573 criminal judgments and case analysis, this study delineates the judicially visible structure of healthcare corruption in China rather than its true prevalence. Evidence shows persistent concentration at key bottlenecks include procurement and supply chain management, medical insurance auditing and payment, and in-hospital financial control, where gatekeeping authority, professional discretion, and fund-interface control may be converted into repeatable rent-seeking arrangements. The central governance implication is not campaign-style punishment alone, but the construction of preventive, transparent, and non-carceral accountability mechanisms that tighten institutional control over rule-entry points, payment-side auditing, and high-risk posts, while linking judicial, administrative, insurance, and organizational supervision systems before corruption becomes entrenched.

## 6 Limitation

Further limitations should be noted. First, the study reflects judicial visibility rather than corruption incidence, due to its reliance on publicly available adjudicative documents. Second, coding based on legal texts offers limited insight into motivations, organizational processes, or transaction networks. Third, typical cases are context-dependent and should not be overgeneralized. Future research should integrate audit, insurance supervision, and procurement-platform data to strengthen external validity. Accordingly, annual distributions are presented as publication-year patterns of judicial visibility, not as incidence estimates or policy-effect measures.

## Data Availability

Publicly available datasets were analyzed in this study. This data can be found here: China Judgments Online.

## References

[1] Vian T. Review of corruption in the health sector: theory, methods and interventions. Health Policy Plan. (2008) 23:83–94. doi: 10.1093/heapol/czm048, 18281310

[2] Hutchinson E Balabanova D McKee M. We need to talk about corruption in health systems. Int J Health Policy Manag. (2019) 8:191–4. doi: 10.15171/ijhpm.2018.123, 31050963 PMC6499907

[3] Jain A Nundy S Abbasi K. Corruption: medicine’s dirty open secret. BMJ. (2014) 348:g4184. doi: 10.1136/bmj.g4184, 24965786

[4] Mackey TK Vian T Kohler J. The sustainable development goals as a framework to combat health-sector corruption. Bull World Health Organ. (2018) 96:634–43. doi: 10.2471/BLT.18.209502, 30262945 PMC6154071

[5] Fu H Lai Y Li Y Zhu Y Yip W. Understanding medical corruption in China: a mixed-methods study. Health Policy Plan. (2023) 38:496–508. doi: 10.1093/heapol/czad015, 36798965

[6] Radin D. Does corruption undermine trust in health care? Results from public opinion polls in Croatia. Soc Sci Med. (2013) 98:46–53. doi: 10.1016/j.socscimed.2013.08.033, 24331881

[7] Rispel LC de Jager P Fonn S. Exploring corruption in the South African health sector. Health Policy Plan. (2016) 31:239–49. doi: 10.1093/heapol/czv047, 26104821

[8] General Office of the State Council (2019) Notice of the General Office of the State Council on issuing the pilot program for the centralized procurement and use of drugs organized by the State. Available online at: https://www.gov.cn/zhengce/content/2019-01/17/content_5358604.htm (Accessed February 16, 2025)

[9] Shi J Liu R Jiang H Wang C Xiao Y Liu N . Moving towards a better path? A mixed-method examination of China’s reforms to remedy medical corruption from pharmaceutical firms. BMJ Open. (2018) 8:e018513. doi: 10.1136/bmjopen-2017-018513, 29439069 PMC5829841

[10] Gorodensky A Bowra A Saeed G Kohler J. Anti-corruption in global health systems: using key informant interviews to explore anti-corruption, accountability and transparency in international health organisations. BMJ Open. (2022) 12:e064137. doi: 10.1136/bmjopen-2022-064137, 36549737 PMC9772658

[11] Yuan S. China’s crackdown on health-care corruption. Lancet. (2023) 402:1234–5. doi: 10.1016/S0140-6736(23)01785-3, 37634512

[12] China News Service. (2025) 2025 anti-corruption storm in medicine and healthcare continues to deepen. Available online at: https://m.chinanews.com/wap/detail/chs/zw/10352476.shtml (Accessed February 16, 2025)

[13] Supreme People’s Procuratorate of China. More than 4,700 people prosecuted for medical insurance fraud crimes in 2024. Available online at: https://www.spp.gov.cn/xwfbh/wsfbt/202501/t20250123_680494.shtml#1 (Accessed February 17, 2025)

[14] CPC Central Commission for Discipline Inspection and National Commission of Supervision (2024) Report on the governance of misconduct and corruption around the people. Available online at: https://www.ccdi.gov.cn/toutiaon/202412/t20241226_396507.html (Accessed February 16, 2025)

[15] Liebman BL Roberts ME Stern RE Wang AZ. Mass digitization of Chinese court decisions: how to use text as data in the field of Chinese law. J Law Courts. (2020) 8:177–201. doi: 10.1086/709916

[16] Su Z Bentley BL Yu X Jiang J Liu Y McDonnell D . Where is the money? Insights into China’s post-COVID healthcare corruption-busting campaign. J Public Health Policy. (2024) 45:396–400. doi: 10.1057/s41271-024-00474-5, 38548971

[17] O’Cathain A Murphy E Nicholl J. The quality of mixed methods studies in health services research. J Health Serv Res Policy. (2008) 13:92–8. doi: 10.1258/jhsrp.2007.007074, 18416914

[18] Bernal JL Cummins S Gasparrini A. Erratum in: Interrupted time series regression for the evaluation of public health interventions: a tutorial. Int J Epidemiol (2017) 46: dyw098–dyw355. doi: 10.1093/ije/dyw098. Int J Epidemiol. (2020) 49:1414. doi: 10.1093/ije/dyaa118, 32879971 PMC7750921

[19] National Healthcare Security Administration (2019) Opinions on expanding the regional scope of the national centralized drug procurement and use pilot. Available online at: https://www.nhsa.gov.cn/art/2019/9/30/art_37_1817.html (Accessed February 16, 2025)

[20] Baxter P Jack S. Qualitative case study methodology: study design and implementation for novice researchers. Qual Rep. (2008) 13:544–59. doi: 10.46743/2160-3715/2008.1573

[21] Fetters MD Curry LA Creswell JW. Achieving integration in mixed methods designs-principles and practices. Health Serv Res. (2013) 48:2134–56. doi: 10.1111/1475-6773.12117, 24279835 PMC4097839

[22] von Elm E Altman DG Egger M Pocock SJ Gøtzsche PC Vandenbroucke JP. The strengthening the reporting of observational studies in epidemiology (STROBE) statement: guidelines for reporting observational studies. J Clin Epidemiol. (2008) 61:344–9. doi: 10.1016/j.jclinepi.2007.11.008, 18313558

[23] Tong A Sainsbury P Craig J. Consolidated criteria for reporting qualitative research (COREQ): a 32-item checklist for interviews and focus groups. Int J Qual Health Care. (2007) 19:349–57. doi: 10.1093/intqhc/mzm042, 17872937

[24] Benchimol EI Smeeth L Guttmann A Harron K Moher D Petersen I . The reporting of studies conducted using observational routinely-collected health data (RECORD) statement. PLoS Med. (2015) 12:e1001885. doi: 10.1371/journal.pmed.1001885, 26440803 PMC4595218

[25] Ran Y Hu Y Chen S Qiu F Rabeeu A. The impact of two-invoice system on pharmaceutical manufacturers’ selling expenses in China: a difference-in-differences approach. Int J Environ Res Public Health. (2022) 19:4400. doi: 10.3390/ijerph19074400, 35410081 PMC8999028

[26] Yuan J Lu ZK Xiong X Jiang B. Lowering drug prices and enhancing pharmaceutical affordability: an analysis of the national volume-based procurement (NVBP) effect in China. BMJ Glob Health. (2021) 6:e005519. doi: 10.1136/bmjgh-2021-005519, 34518200 PMC8438819

[27] Wang J Yang Y Xu L Shen Y Wen X Mao L . Impact of “4 + 7” volume-based drug procurement on the use of policy-related original and generic drugs: a natural experimental study in China. BMJ Open. (2022) 12:e054346. doi: 10.1136/bmjopen-2021-054346, 35288385 PMC8921850

[28] Wang X He X Zhang P Zhang M Ma R Dai R . The impact of the national volume-based procurement policy on the use of policy-related drugs in Nanjing: an interrupted time-series analysis. Int J Equity Health. (2023) 22:200. doi: 10.1186/s12939-023-02006-1, 37770924 PMC10540346

[29] National Health Commission of China (2024) Notice on issuing the 2024 work plan for rectifying unhealthy practices in pharmaceutical procurement and sales and in medical services. Available online at: https://www.nhc.gov.cn/ylyjs/zcwj/202405/ad816c4685ef4dcfb475554d12241afa.shtml (Accessed February 16, 2025)

[30] General Office of the State Council of China (2024) Notice of the General Office of the State Council on issuing the key tasks for deepening health care reform in 2024. Available online at: https://www.nhc.gov.cn/tigs/c100053/202406/812e38d8a1bf4f72a2cdabd8a62ebed4.shtml (Accessed February 16, 2025)

[31] National Healthcare Security Administration of China (2024) Notice on conducting 2024 medical insurance fund “flying inspections”. Available online at: https://www.nhsa.gov.cn/art/2024/4/28/art_104_12528.html (Accessed February 16, 2025)

[32] Stepurko T Pavlova M Gryga I Groot W. Empirical studies on informal patient payments for health care services: a systematic and critical review of research methods and instruments. BMC Health Serv Res. (2010) 10:273. doi: 10.1186/1472-6963-10-273, 20849658 PMC2955014

[33] Li H Li L Liu T Tan M He W Luo Y . Risk management and empirical study of the doctor-patient relationship: based on 1790 litigation cases of medical damage liability disputes in China. BMC Health Serv Res. (2024) 24:521. doi: 10.1186/s12913-024-10952-x, 38664671 PMC11044444

[34] Sun J Shen S Han Q Zhang L. Patient influencing doctor-patient dispute trials: evidence from China. Front Psychol. (2026) 17:1734171. doi: 10.3389/fpsyg.2026.1734171, 41694021 PMC12900752

[35] Mackey TK Liang BA. Combating healthcare corruption and fraud with improved global health governance. BMC Int Health Hum Rights. (2012) 12:23. doi: 10.1186/1472-698X-12-23, 23088820 PMC3519514

[36] Vian T. Anti-corruption, transparency and accountability in health: concepts, frameworks, and approaches. Glob Health Action. (2020) 13:1694744. doi: 10.1080/16549716.2019.1694744, 32194010 PMC7170369

[37] World Health Organization (2023) Tackling corruption to move towards universal health coverage and health security. Available online at: https://cdn.who.int/media/docs/default-source/universal-health-coverage/who-uhl-technical-brief-anti-corruption.pdf?download=true&sfvrsn=a9a746f2_3l (Accessed February 16, 2025)

[38] Shleifer A Vishny RW. Corruption. Q J Econ. (1993) 108:599–617. doi: 10.2307/2118402

[39] Bandiera O Prat A Valletti T. Active and passive waste in government spending: evidence from a policy experiment. Am Econ Rev. (2009) 99:1278–308. doi: 10.1257/aer.99.4.1278

[40] Arrow KJ. Uncertainty and the welfare economics of medical care. 1963. J Health Polit Policy Law. (2001) 26:851–83. doi: 10.1215/03616878-26-5-851, 11765268

[41] Kohler JC Dimancesco D. The risk of corruption in public pharmaceutical procurement: how anti-corruption, transparency and accountability measures may reduce this risk. Glob Health Action. (2020) 13:1694745. doi: 10.1080/16549716.2019.1694745, 32194011 PMC7170361

[42] Mugellini G Della Bella S Colagrossi M Isenring GL Killias M. Public sector reforms and their impact on the level of corruption: a systematic review. Campbell Syst Rev. (2021) 17:e1173. doi: 10.1002/cl2.1173, 37131927 PMC8356278

[43] Di Tella R Schargrodsky E. The role of wages and auditing during a crackdown on corruption in the city of Buenos Aires. J Law Econ. (2003) 46:269–92. doi: 10.1086/345578

[44] Ferraz C Finan F. Electoral accountability and corruption: evidence from the audits of local governments. Am Econ Rev. (2011) 101:1274–311. doi: 10.1257/aer.101.4.1274

[45] Wazana A. Physicians and the pharmaceutical industry: is a gift ever just a gift? JAMA. (2000) 283:373–80. doi: 10.1001/jama.283.3.373 10647801

[46] Spurling GK Mansfield PR Montgomery BD Lexchin J Doust J Othman N . Information from pharmaceutical companies and the quality, quantity, and cost of physicians’ prescribing: a systematic review. PLoS Med. (2010) 7:e1000352. doi: 10.1371/journal.pmed.1000352, 20976098 PMC2957394

[47] DeJong C Aguilar T Tseng CW Lin GA Boscardin WJ Dudley RA. Pharmaceutical industry-sponsored meals and physician prescribing patterns for Medicare beneficiaries. JAMA Intern Med. (2016) 176:1114–22. doi: 10.1001/jamainternmed.2016.2765, 27322350

[48] Goupil B Balusson F Naudet F Esvan M Bastian B Chapron A . Association between gifts from pharmaceutical companies to French general practitioners and their drug prescribing patterns in 2016: retrospective study using the French transparency in healthcare and National Health Data System databases. BMJ. (2019) 367:l6015. doi: 10.1136/bmj.l6015, 31690553 PMC6830500

[49] Mitchell AP Trivedi NU Gennarelli RL Chimonas S Tabatabai SM Goldberg J . Are financial payments from the pharmaceutical industry associated with physician prescribing? A systematic review. Ann Intern Med. (2021) 174:353–61. doi: 10.7326/M20-5665, 33226858 PMC8315858

[50] Iwai Y Khan ZH DasGupta S. Abolition medicine. Lancet. (2020) 396:158–9. doi: 10.1016/S0140-6736(20)31566-X, 32682471 PMC7365639

[51] Deivanayagam TA Lasoye S Smith J Selvarajah S. Policing is a threat to public health and human rights. BMJ Glob Health. (2021) 6:e004582. doi: 10.1136/bmjgh-2020-004582, 33547177 PMC7871237

[52] Zovic HL Riley T Perez-Bill ES Dsouza N Mitchell C. A call for a transformative public health training: the necessity of abolition. Health Educ Behav. (2023) 50:465–72. doi: 10.1177/10901981231177085, 37525984 PMC11075666

