# Correction: Reforming the white coat economy: judicial evidence and institutional implications from China's healthcare anti-corruption campaign

**DOI:** 10.3389/fpubh.2026.1887748

**Published:** 2026-06-05

**Authors:** Frontiers Production Office

**Affiliations:** Frontiers Media SA, Lausanne, Switzerland

**Keywords:** adjudicative document analysis, anti-corruption in healthcare, healthcare corruption, judicial evidence, legal mechanisms, white coat economy

Various specific numerical values were erroneously published in their original, unblurred form, with one instance of repetition.

A correction has been made to the section **3.2 In-depth depiction of typical cases**, sub-section “*3.2.1 The institutional-gatekeeper type*”, Paragraph 2:

“The court also identified additional distributions to “meeting members” totaling a six-figure RMB sum (Supplementary Appendix S2, Case 1).”

A correction has been made to the section **3.2 In-depth depiction of typical cases**, sub-section “*3.2.1 The institutional-gatekeeper type*”, Paragraph 4:

“In one compensation case, actors used internal documents to establish formal identities and unilaterally adjust pay standards, enabling an individual to receive high compensation for 7 years without performing duties, with cumulative embezzlement of a six-figure RMB sum (Supplementary Appendix S2, Case 3). In another case, drug and consumable rebates totaling an eight-figure RMB sum were absorbed as a shadow budget under labels such as “net profit awards” and “consultation fees,” alongside abuse of power resulting in state losses of a seven-figure RMB sum and secondary collective embezzlement (Supplementary Appendix S2, Case 4).”

A correction has been made to the section **3.2 In-depth depiction of typical cases**, sub-section “*3.2.2 The external supplier type*”, Paragraph 3:

“The same mechanism appeared in construction and project bidding. A contractor cultivated a hospital director and spouse with low visibility gifts over 8 years (such as New Year visits and birthdays), totaling a six-figure RMB sum, before winning an interior decoration contract worth nearly forty million RMB (Supplementary Appendix S2, Case 6).”

A correction has been made to the section **3.2 In-depth depiction of typical cases**, sub-section “*3.2.3 The funds-handler type*”, Paragraph 2:

“In one case, a branch cashier forged seals and deposit records to divert collected funds, embezzling a seven-figure RMB sum from rental income, utility fees, and patient charges (Supplementary Appendix S2, Case 7).”

Another correction has been made to the section **3.2 In-depth depiction of typical cases**, sub-section “*3.2.3 The funds-handler type*”, Paragraph 2:

“The audit found that falsely claimed pharmaceuticals alone accounted for a six-figure RMB sum (Supplementary Appendix S2, Case 8).”

A correction has been made to the section **3.2 In-depth depiction of typical cases**, sub-section “*3.2.3 The funds-handler type*”, Paragraph 3:

“System vulnerabilities may reinforce this pattern. In one case, a billing staff member exploited a system weakness allowing improper payment-method selection, recording cash payments as “check payments” and intercepting a substantial amount of cash, the bulk of which remained outstanding for more than three months. (Supplementary Appendix S2, Case 9).”

A correction has been made to the section **3.2 In-depth depiction of typical cases**, sub section “*3.2.4 The technical adjudicator type*”, Paragraph 2:

“A second form is the privatized monetization of medical technology operating rights. An MRI physician bypassed the hospital billing system, privately charging for over a thousand body parts, collecting several hundred thousand RMB, and causing nearly six hundred thousand RMB in lost revenue. (Supplementary Appendix S2, Case 11).”

A correction has been made to the section **3.2 In-depth depiction of typical cases**, sub section “*3.2.4 The technical adjudicator type*”, Paragraph 4:

“Multiple clinicians implemented “yin-yang prescriptions” and assisted reimbursement, involving defrauded amounts ranging from hundreds of thousands to over one million RMB.”

The original version of this article has been updated.

