# Supplementary material for: Reforming the white coat economy: judicial evidence and institutional implications from China’s healthcare anti-corruption campaign
Source: Front Public Health. 2026 May 18;14:1818452. doi: 10.3389/fpubh.2026.1818452 (PMC13223165; doi:10.3389/fpubh.2026.1818452)
Supplement: Supplementary file 1 [file Data_sheet_1.pdf]

Appendix S1. Search strategy and keyword dictionaries

This appendix consolidates the offence list and keyword dictionaries used for stepwise retrieval of criminal judgments related to medical-sector corruption on China Judgments Online (CJO). The operational search terms are provided in both English and Chinese to support transparency and reproducibility.

1. Offence list for the “offence/cause-of-action” pathway

Use the offence/cause-of-action filters for offence-by-offence searches. It is recommended to run one search per offence and record the number of hits.

1.1 Core bribery-related offences

Acceptance of bribes (受贿罪)

Acceptance of bribes by a unit (单位受贿罪)

Offering bribes (行贿罪)

Offering bribes by a unit (单位行贿罪)

Offering bribes to a unit (对单位行贿罪)

Offering bribes to persons with influence / Acceptance of bribes by using influence (对有影响力的人行贿罪 / 利用影响力受贿罪)

Acceptance of bribes by non-state functionaries (非国家工作人员受贿罪)

Offering bribes to non-state functionaries (对非国家工作人员行贿罪)

Introducing bribery (介绍贿赂罪)

1.2 Extended offences potentially relevant to medical corruption

These offences may be considered when the operational definition of “medical corruption” extends beyond bribery to include embezzlement/misappropriation and insurance or procurement fraud.

Embezzlement (贪污罪 (including cases involving medical institutions or public institutions))

Misappropriation of public funds (挪用公款罪)

Embezzlement by an employee (occupational embezzlement) (职务侵占罪 (may appear in some private hospitals or corporatized entities))

Contract fraud / Fraud (合同诈骗罪 / 诈骗罪 (e.g., involving medical insurance funds or procurement contracts where the facts indicate an “interest-transfer to fraudulent extraction” chain))

Note: Whether to include the extended offences depends on the operational definition of “medical corruption” (e.g., limited to bribery/acceptance of bribes versus also covering embezzlement, misappropriation, and insurance fraud). Some prior studies have adopted a broader scope (bribery + embezzlement + insurance fraud)[1].

2. Keyword dictionaries (Chinese search terms)

The following dictionaries were used as full-text/summary keywords. Terms are listed with English glosses and their Chinese forms in parentheses.

2.1 Institutions and care settings

hospital (医院)

township hospital (卫生院)

community health service center (社区卫生服务中心)

outpatient clinic (门诊)

emergency department (急诊)

inpatient ward (住院部)

operating room (手术室)

intensive care unit (ICU)

medical affairs department (医务科)

pharmacy department (药剂科)

equipment department (设备科)

procurement office (采购办)

tendering and procurement office (招采办)

logistics department (后勤)

capital construction/infrastructure (基建)

laboratory department (检验科)

radiology/imaging department (影像科)

pathology department (病理科)

information technology department (信息科)

2.2 Personnel and roles

doctor/physician (医生)

physician (医师)

chief physician (主任医师)

department head (科主任)

hospital president/director (院长)

vice president/deputy director (副院长)

head nurse (护士长)

pharmacist (药师)

laboratory technologist (检验师)

head of equipment office/department (设备处/科负责人)

procurement officer (采购员)

tendering officer (招标负责人)

2.3 Corruption-behaviour keywords

hongbao (cash gift / under-the-table payment) (红包)

kickback (回扣)

commission (提成)

rebate (返利)

benefit fee (好处费)

gratitude fee (感谢费)

service fee (in anomalous contexts) (劳务费（异常语境）)

consulting fee (in anomalous contexts) (咨询费（异常语境）)

sponsorship fee (赞助费)

academic conference fee (学术会议费)

lecture fee (in anomalous contexts) (讲课费（异常语境）)

gift money (礼金)

gifts (礼品)

shopping/gift card (购物卡)

cash (现金)

bank transfer (转账)

rebate/return point (返点)

commission (佣金)

intermediary fee (居间费)

agency/brokerage fee (中介费)

2.4 Procurement and supply-chain keywords

pharmaceuticals/drugs (药品)

medical consumables (耗材)

high-value medical consumables (高值耗材)

medical devices/instruments (器械)

equipment (设备)

reagents (试剂)

laboratory testing (检验)

test reagents (检测试剂)

supply (供货)

supplier (供应商)

distributor (经销商)

agent (代理商)

pharmaceutical sales representative (医药代表)

distribution/delivery (配送)

platform listing (procurement platform) (挂网)

volume-based procurement (带量采购)

tendering (招标)

bidding (投标)

bid award (中标)

price negotiation (议价)

procurement/purchasing (采购)

contract (合同)

acceptance/inspection (验收)

warehousing/stock-in (入库)

payment collection/receivables (回款)

invoice (发票)

3. Recommended query sets

Set 1 (primary, precise)

Fields: Case type = criminal; Offence/cause-of-action = select one offence from the offence list; Full text or summary = select 1-3 medical-context keywords.

Set 2 (supplementary, to capture non-typical offences with clear medical context)

Fields: Case type = criminal; Full text or summary = corruption-behaviour keywords + medical-context keywords.

Set 3 (supply-chain linkage, to locate the procurement-supplier interface)

Fields: Case type = criminal; Full text or summary = procurement/supply-chain keywords + corruption-behaviour keywords + medical-context keywords.

Set 4 (only when using an expanded offence scope)

Fields: Case type = criminal; Offence/cause-of-action = embezzlement/misappropriation/occupational embezzlement, etc.; Full text or summary = hospital/drugs/consumables/equipment/tendering[2][3].

4. References

[1] Fu H, Lai Y, Li Y, Zhu Y, Yip W. Understanding medical corruption in China: a mixed-methods study. *Health Policy Plan*. 2023;38(4):496-508. doi:10.1093/heapol/czad015

[2] Shi J, Liu R, Jiang H, et al. Moving towards a better path? A mixed-method examination of China's reforms to remedy medical corruption from pharmaceutical firms. *BMJ Open*. 2018;8(2):e018513. Published 2018 Feb 8. doi:10.1136/bmjopen-2017-018513

[3] Liebman BL, Roberts ME, Stern RE, Wang AZ. Mass Digitization of Chinese Court Decisions: How to Use Text as Data in the Field of Chinese Law. J Law Courts. 2020;8:177-223.
