# Supplementary material for: Reforming the white coat economy: judicial evidence and institutional implications from China’s healthcare anti-corruption campaign
Source: Front Public Health. 2026 May 18;14:1818452. doi: 10.3389/fpubh.2026.1818452 (PMC13223165; doi:10.3389/fpubh.2026.1818452)
Supplement: Supplementary file 3 [file Data_sheet_3.docx]

***Supplementary Material***

**1 Supplementary Figures**


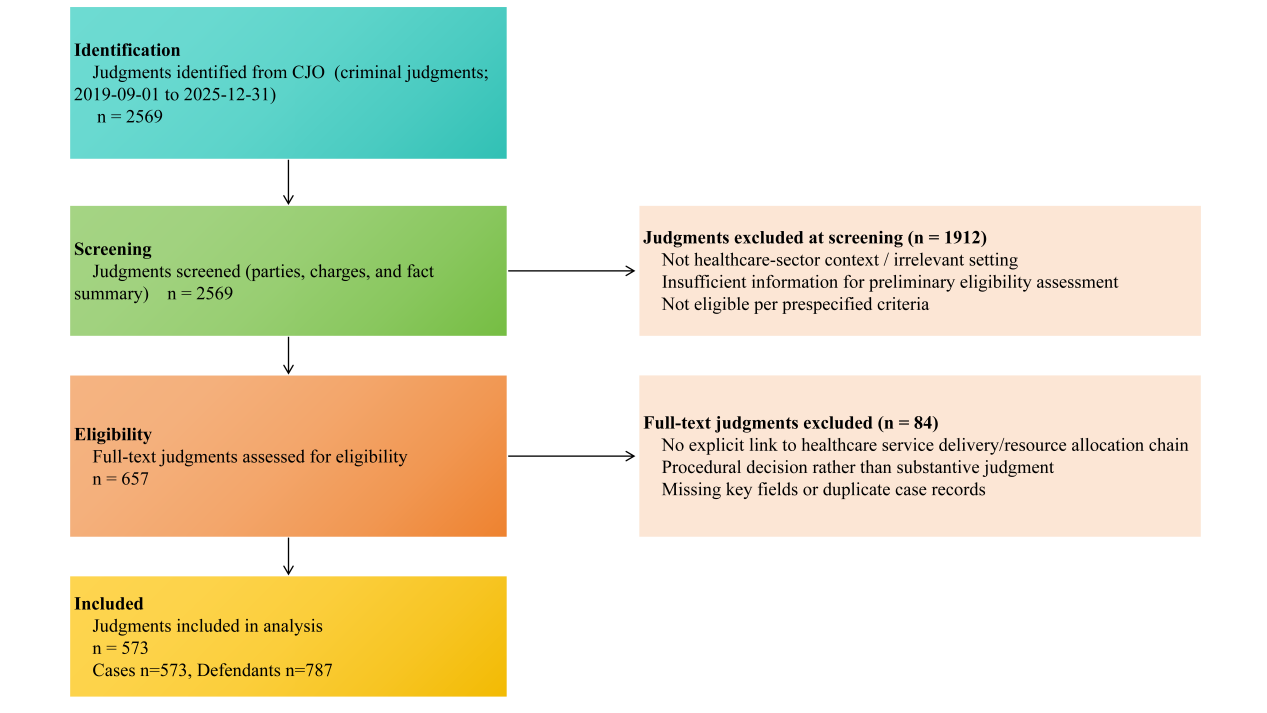


**Figure 1. Identification, screening, eligibility assessment, and inclusion of CJO criminal judgments related to healthcare-sector corruption, 2019.12–2025.**

**2 Supplementary Tables**

Table 1 Basic Characteristics of the Sample, Types of Corruption, and Governance Risk Points

| **Indicators** | **n** | **%** |
| --- | --- | --- |
| **A.Time Distribution (by Judgment Year)** | **573** | **100%** |
| 2019 (Sep–Dec) | 158 | 27.57% |
| 2020 | 180 | 31.41% |
| 2021 | 58 | 10.12% |
| 2022 | 20 | 3.49% |
| 2023 | 21 | 3.66% |
| 2024 | 57 | 9.95% |
| 2025 | 79 | 13.79% |
| **B.Region** | **573** | **100%** |
| Eastern China | 131 | 22.86% |
| Central China | 188 | 31.81% |
| Western China | 205 | 35.78% |
| Northeast China | 49 | 8.55% |
| **C.Court Level** | **573** | **100%** |
| Primary Court | 534 | 93.19% |
| Intermediate Court | 37 | 6.46% |
| High Court | 1 | 0.17% |
| Specialized Court | 1 | 0.17% |
| **D.Main Types of Medical Corruption (Mutually Exclusive Primary Categories)** | **573** | **100%** |
| Pharmaceutical Sales/Clinical Commercial Bribery | 267 | 46.60% |
| Embezzlement of In-Hospital Funds (Corruption/Embezzlement/Misappropriation of Funds) | 137 | 23.91% |
| Fraud (False Reimbursement of Medical Insurance Funds, Impersonation) | 99 | 17.28% |
| Bribery of Non-State Functionaries/Bribery to Non-State Functionaries/Corporate Bribery | 45 | 7.85% |
| Bid Rigging | 25 | 4.36% |
| **E.Governance Risk Points (Multiple Selection Possible, Counted Based on Whether the Case Involves This Link)** | **/** | **/** |
| Procurement and Supply Chain (Pharmaceuticals/Consumables/Equipment/Informatization/Construction/Business Cooperation and Outsourcing, etc.) | 335 | / |
| Medical Insurance Audit and Payment (Settlement/Audit/Inspection/Impersonation, etc.) | 136 | / |
| In-Hospital Finance and Asset Management (Budget, Billing, Reimbursement, Bills/Notes, Asset Disposal, etc.) | 122 | / |
| Administrative Supervision and Resource Allocation (Access Qualification/Fund Allocation, Personnel Management, etc.) | 61 | / |

Note: Cases may involve multiple governance risk points; therefore, the sum of counts across risk points exceeds the total number of cases (n=573).

Table 2 Key Actors and Transaction Pathways

| **Indicators** | **n** | **%** |
| --- | --- | --- |
| **A. Actors Involved** | **787** | **100%** |
| Hospital Leadership (Presidents/Party Secretaries/Deputy Heads) | 223 | 28.34% |
| Third-Party Service Providers (Suppliers of medical devices, pharmaceuticals, consumables; construction and engineering contractors, etc.) | 141 | 17.92% |
| Personnel in Key Positions (Accountants, Billing Clerks, Cashiers, etc.) | 114 | 14.49% |
| Heads of Administrative/Functional Departments (Finance/Procurement/Equipment/Logistics, etc.) | 99 | 12.58% |
| Clinical Medical and Nursing Staff | 88 | 11.18% |
| Heads of Clinical/Medical Technology Departments | 59 | 7.5% |
| Health Administration Officials and Heads of Relevant State-Owned Enterprises/Institutions | 45 | 5.72% |
| Others (Patients/Intermediaries/Specified Related Persons) | 14 | 1.78% |
| Hospitals and Their Internal Departments (as institutional actors) | 4 | 0.51% |
| **B. Actors with Entrusted Power** | **145** | **100%** |
| Hospital Leadership (Presidents/Party Secretaries/Deputy Heads, including those at township health centers and private hospitals) | 65 | 44.83% |
| Heads of Administrative/Functional Departments (Finance/Procurement/Equipment/Logistics, etc.) | 40 | 27.59% |
| Heads of Medical Technology/Clinical Departments | 19 | 13.10% |
| Clinical Medical and Nursing Staff | 12 | 8.28% |
| Health Administration Officials and Heads of Relevant State-Owned Enterprises/Institutions | 7 | 4.83% |
| Personnel in Key Positions (Accountants, Billing Clerks, Cashiers, etc.) | 2 | 1.38% |
| 1. **Transaction Pathways (Top 3)** | **/** | **/** |
| Cash/Bank Transfers/Real Estate/Vehicles/Gifts & Cash Equivalents | 272 | / |
| Kickbacks/Rebates/Commissions | 116 | / |
| Fraudulent Contracts/Disguised Dividends/Disguised Payments ("Training Fees", "Service Fees", "Interest", "Loans", etc.) | 53 | / |

Table 3 Judicial Dispositions and Discretionary Factors

| **Primary**  **Case Type** | **Defendants (n)** | **Median Principal Penalty (months)** | **Probation (%)** | **Fine(%)** | **Recovery/Disgorgement/Forfeiture (%)** | **Guilty Plea and Acceptance of Penalty (%)** | **Voluntary Surrender/Meritorious Service/Frank Confession (%)** |
| --- | --- | --- | --- | --- | --- | --- | --- |
| Bribery (Active and Passive) | 268 | 36(30) | 28.73% | 96.64% | 83.58% | 85.07% | 94.4% |
| Fraud | 202 | 36(23) | 61.39% | 100% | 84.65% | 75.74% | 86.14% |
| Corruption / Embezzlement / Misappropriation | 173 | 36(38) | 28.9% | 74.57% | 92.49% | 78.03% | 87.86% |
| Bid Rigging | 30 | 9.5(5.75) | 86.67% | 100% | 70% | 86.87% | 100% |
| Commercial Bribery (Non-State Actor) / Bribery of Non-State Functionaries | 34 | 17.5(13.75) | 79.41% | 76.47% | 79.41% | 91.18% | 85.29% |
| Corporate Bribery | 3 | 30(10) | 33.33% | 100% | 0 | 100% | 66.67% |
| Total | 710 | 36(30) | 42.90% | 91.70% | 84.81% | 81.43% | 89.73% |

Note: This table is based on defendants sentenced to fixed-term imprisonment (n = 710). Defendants not sentenced to fixed-term imprisonment (e.g., sentenced to criminal detention, public surveillance, exempted from criminal punishment, or subject only to fines, etc.) are excluded from this table.

Table 4 Typology of Key Actors in Medical Corruption

| **Type** | **Scope of Subjects** | **Basis of Authority** | **Direction of Acts** | **Primary Areas Involved** | **Essential Characteristics** |
| --- | --- | --- | --- | --- | --- |
| Institutional Gatekeepers | Hospital Leadership, Heads of Administrative/Functional Departments, Health Administration Personnel | Statutory Authority, Organizational Delegation | External Approval/Internal Management | Resource Allocation Arena (Procurement Initiation/Budget Approval/Personnel Appointment/Rule-Making) | Highly Concentrated Decision-Making Power, Broad Discretionary Scope, Procedural Constraints Primarily Dependent on Internal Processes |
| External Suppliers | Medical Device/Pharmaceutical/Consumables Suppliers, Third-Party Service Providers, Patients, Intermediaries | Market Position, Commercial Contract | Transfer of Benefits into the Institutional System | Market-Institution Interface Arena (Bidding/Supply/Agent/Contracting) | Transacting Parties Operate Under Distinct Logics—Institutional Sector Pursues Compliance and Stability, Market Sector Pursues Share and Profit |
| Funds Handlers | Personnel in Key Positions (Accountants/Cashiers/Billing Clerks) \| Division of Functions, Operational Authorization | Internal Appropriation | Internal Appropriation | Funds Settlement Arena (Billing/Cashiering/Reconciliation/Invoice Verification) | High-Frequency Repetitive Transactions, Dispersed Individual Amounts, Control Dependent on System Settings |
| Technical Adjudicators | Heads of Clinical/Medical Technology Departments, Clinical Medical and Nursing Staff \| Professional Knowledge, Clinical Discretion | Professional Knowledge, Clinical Discretion | Internal Adjudication/Internal-External Collusion in Treatment Plans/Consumables Selection | Clinical Service Arena (Prescribing/Consumables Selection/Surgical Determination/Admission Indications) | High Professional Barriers, Discretion Embedded in Clinical Logic, External Supervision Difficult to Penetrate |
